# Isolated myeloid sarcoma of the temporal bone: As the first clinical manifestation of acute myeloid leukemia in a patient of down’s syndrome

**DOI:** 10.1016/j.ijscr.2019.03.027

**Published:** 2019-03-28

**Authors:** Nisha Marwah, Namita Bhutani, Archana Budhwar, Rajeev Sen

**Affiliations:** Dept. of Pathology, PGIMS Rohtak, Haryana, India

**Keywords:** Acute myeloid leukemia, Down syndrome, Extramedullary, Granulocytic sarcoma, Myeloid sarcoma

## Abstract

**Introduction:**

Leukemia is the most common malignancy of childhood but myeloid sarcoma is a rare presentation of underlying leukemic disorder. Myeloid sarcoma (MS) is a rare tumor composed of proliferation of myeloid precursors at extramedullary sites.

**Presentation of case:**

We report an unusual case of myeloid sarcoma involving the temporal bone in a young male child who presented with a large mass involving the left temporal region. This lesion was the initial presentation which led to further diagnosis of acute myeloid leukemia in our case. This case report brings awareness to the diverse extramedullary manifestations of isolated myeloid sarcoma, as well as the importance and difficulties that are associated with establishing a rapid diagnosis and initiating treatment.

**Discussion:**

They can arise de novo or in association with hematological malignancies, most commonly acute myeloid leukemia (AML-M2). Clinically, it can masquerade as an abscess, cutaneous ulcer, or as a mass lesion. Morphologically, MS can mimic a variety of small round cell tumors including lymphomas, neuroblatoma and rhabdomyosarcoma. The occurrence of this tumor usually heralds AML or the onset of the blastic phase of chronic myeloid leukemia. Early recognition of this rare entity is important, because early aggressive chemotherapy and focal irradiation can cause regression of the tumor and thus improve patient longevity.

**Conclusion:**

The possibility of MS should be considered when dealing with unusual lymphoma like neoplasms that cannot be categorized as any of the Non-Hodgkin lymphoma subtypes and small blue round cell tumors.

## Highlights

• MS are extremely rare tumors with an aggressive clinical course.
• Morphologically, they can mimic small round cell tumors. This problem is further complicated by the lack of clinical suspicion in patients without any hematological disorder.
• Accurate diagnosis of isolated MS requires a multifactorial approach including histopathology, immunophenotyping, immunohistochemistry and cytogenic abnormalities.
• A high level of suspicion is necessary, because early, accurate diagnosis is important to avoid delaying appropriate chemotherapy. A delay in the diagnosis may result in unwarranted fatality particularly so in pediatric patients.
• Here, we report an unusual case of MS initially presenting as a tumor of the temporal bone and first manifestation of AML, in a pediatric patient suffering from Down’s syndrome. This case illustrates the challenges associated with offering a rapid diagnosis with an early initiation of treatment.

## 1 Introduction

Myeloid sarcoma (MS) is a rare disease entity. It is also known as granulocytic sarcoma, extramedullary myeloid neoplasm, or chloroma and is a collection of immature myeloid cells at extramedullary sites. First described by Burns in 1811, the term “Chloroma” was coined by King in 1853 to describe this tumor entity based on its green color which is due to the enzyme myeloperoxidase (MPO) present in the myeloid cells [1]. Granulocytic sarcomas occur most frequently in the pediatric population and in 2.5% to 5% of all patients with Acut Myeloid Leukemia, without sex predilection and is most commomly seen in AML-M2 [2]. It occurs most commonly in bone, periosteum, soft tissue, lymph nodes and skin, although it can arise anywhere throughout the body. When located in bone, leukemic cells are thought to originate in the bone marrow and travel via haversian systems to collect in the subperiosteum [3]. It can also arise de-novo, precede, or occur in association with any myelodysplastic syndrome (MDS), myeloproliferative disorder (MPD), or most commonly AML. Given the various sites of occurrence, the clinical manifestations of MS are diverse with the signs and symptoms specific to the location at which it occurs [2]. Symptoms are secondary to the mass effect of the tumor. When the bone marrow (BM) biopsy does not demonstrate any hematological malignancy, the MS is described as nonleukemic or isolated [4]. In patients without any preexisting hematological disorder, the diagnosis of MS is generally delayed due to the lack of suspicion. Morphologically, it can mimic “small round cell tumors” and lymphomas, thus contributing to the diagnostic difficulty. The discovery of this tumor may represent the first sign of AML or relapse or may herald the onset of the blastic phase of chronic myeloid leukemia [2,4]. Although no standard treatment protocol has been established, early aggressive chemotherapy may represent the best chance for remission. However, the long-term prognosis for these patients remains poor. Here, we report an unusual case of MS initially presenting as a tumor of the temporal bone and first manifestation of AML, in a pediatric patient suffering from Down’s syndrome. This case illustrates the challenges associated with offeringa rapid diagnosis with an early initiation of treatment. The SCARE criteria were utilized for this case report [5].

## 2 Case report

**Fig. 1:**
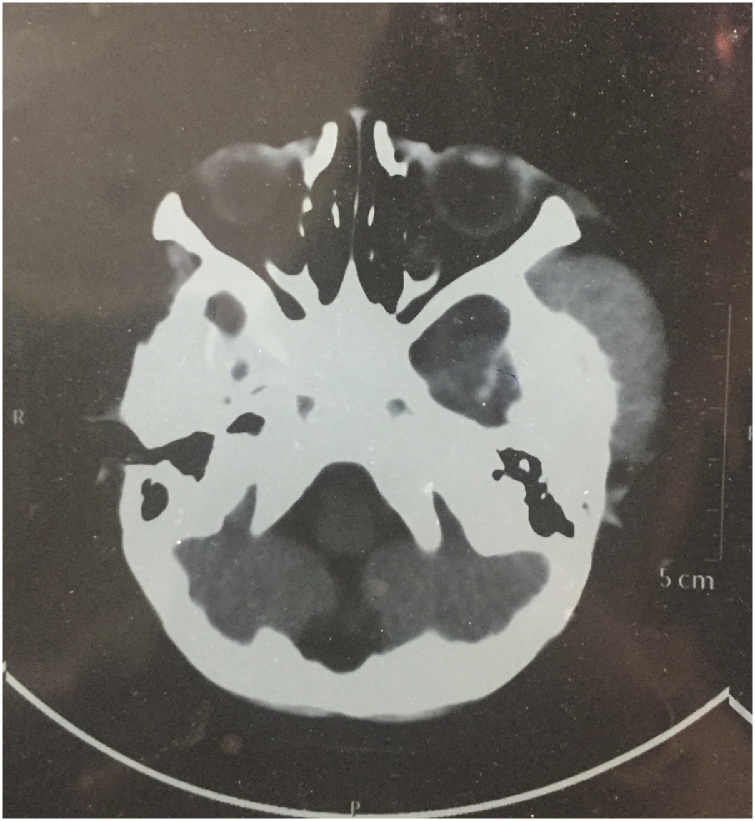
On MRI, a lesion measuring 5 × 3 cm in left temporal region eroding the underlying bone.

A 2 year-old male child was brought to pediatric outpatient department with swelling in temporal region and history of convulsions one week back. For which he was treated by some private practioner and was reffered at our centre for further management. The patient had a history of recent trauma. Considering this, X-Ray was advised, which revealed a mass lesion in left temporal region. Magnetic resonance imaging (MRI) of the head revealed a well-defined 5 cm × 3 cm enhancing lesion with altered signal intensity in the left temporal region. The lesion showed intense contrast enhancement with underlying bone erosion and involving the left cerebellopontine angle (Fig. 1). Imaging findings were suspected of a metastatic lesion. On reviewing the records of the patient, we came to know that patient is a known case of Down’s syndrome.

**Fig. 2:**
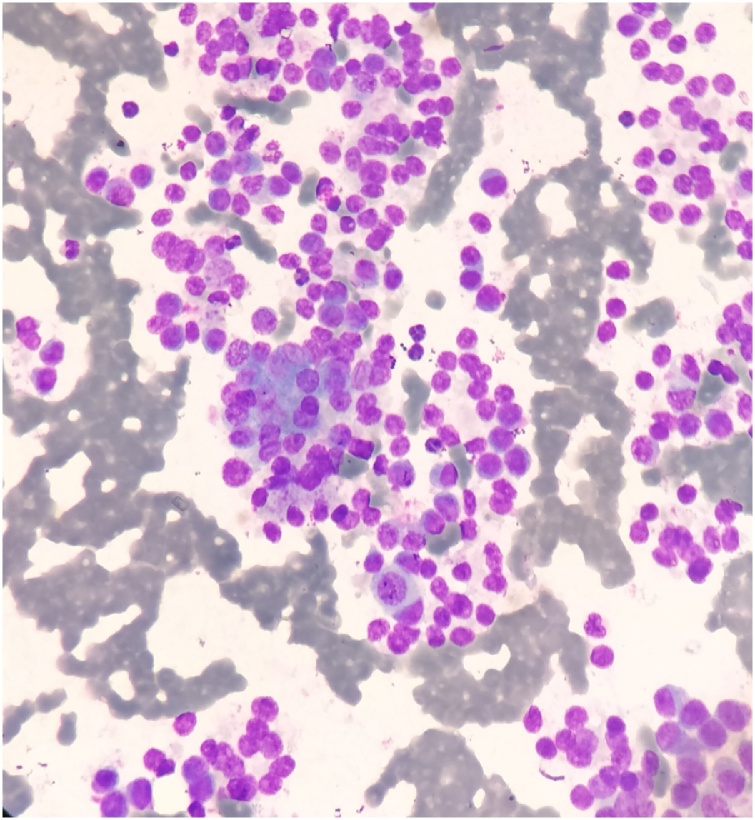
FNAC revealing small, blue, round cells forming rosettes at places (Leishman, 40×).

**Fig. 3:**
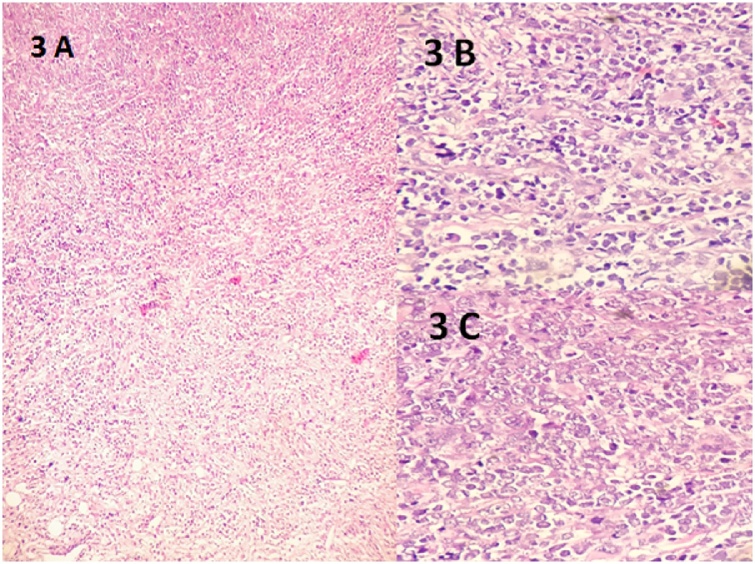
A–C: Myeloid blasts infiltrating the muscles (H & E, A-40×, B-200×, C-400×).

**Fig. 4:**
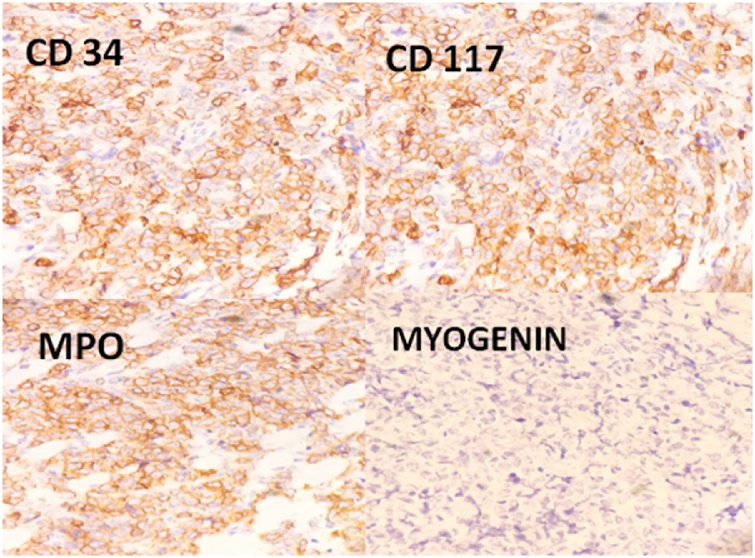
A-D: Tumor cells are positive for MPO, CD34, CD117 and negative for myogenin (200×).

Fine needle aspiration cytology (FNAC) of the mass was performed which showed small, uniform, blue, round cells which at places were forming rosettes (Fig. 2). Considering these findings, a diagnosis of small blue round cell tumor favouring neuroblastoma was rendered. Incisional Biopsy of the mass was performed which revealed a tumor in multiple nodules surrounding the skeletal muscles. Tumor was composed of highly atypical cells, arranged diffusely and in infiltrative pattern in the fibrovascular background. Tumor cells were pleomorphic, round to oval, with vesicular nuclei. Fair number of typical and atypical mitosis was also seen (Fig. 3A–C). On IHC, these cells were strongly and diffusely positive for MPO, CD 117 and CD 34, focally positive for CD 99, vimentin and HLA DR and negative for LCA, CD 20, CD3, CD 10, CD 56, Tdt, CK, NB84 and synaptophysin (Fig. 4A–D). Myogenin and Desmin were negative ruling out possibility of rhabdomyosarcoma. Histopathological diagnosis of myeloid sarcoma was made.

**Fig. 5:**
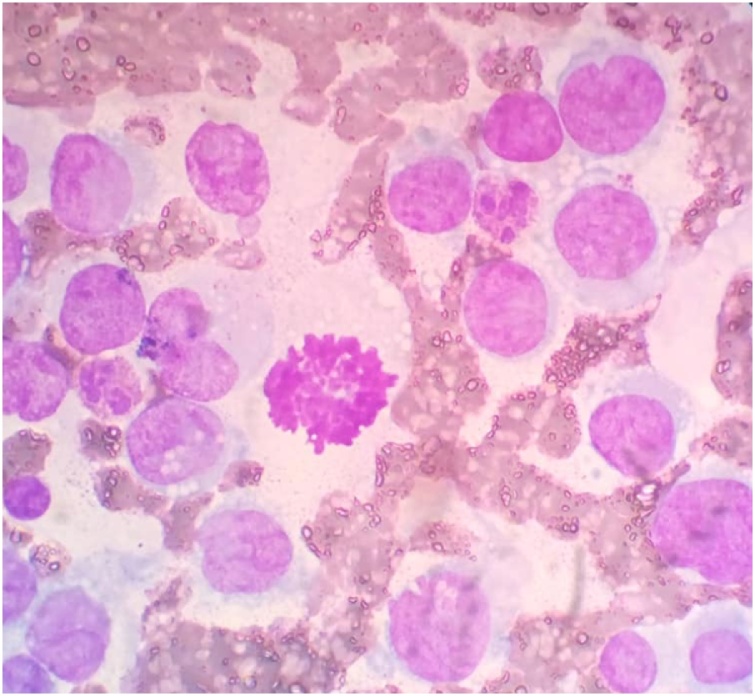
Peripheral blood film demonstrating myeloid blasts with abundant cytoplasm.

Extensive haematological workup, including bone marrow biopsy and immunophenotyping, cytogenetics and positron emission tomography (PET) scan were performed, all yielded negative results and the patient was discharged from hospital without additional treatment and the decision to begin induction chemotherapy with daunorubicin and cytarabine was made. After a month patient returned for his treatment. At that time peripheral blood examination revealed 90% atypical cells/blasts conforming to morphology of myeloid blasts (Fig. 5). Child was admitted for chemotherapy and is on regular follow up. He is doing well and is in complete remission.

## 3 Discussion

Leukemias are the commonest pediatric malignancies with 15%–20% of cases originating from myeloid origin [6]. Patients with myeloid leukemia are prone to develop rare solid tumors of primitive precursors of granulocytic series termed as chloromas or granulocytic sarcomas.Myeloid sarcoma (MS) is a rare, extramedullary collection of primitive granulocyte precursors namely myeloblasts, promyelocytes, and myelocytes along with supporting connective tissue and vascular stroma accounting for 3–8% of the cases, with or without history of leukemia. It is known as chloroma because of its greenish hue due to the presence of intracellular myeloperoxidase.It is most frequently associated with AML-M2, but has also been described in association with other myeloproliferative diseases, like chronic myeloid leukemia (CML), polycythemia vera, hypereosinophilia, and myeloid metaplasia [7]. In a known case of leukemia, development of MS may represent relapse of an already treated systemic disease or may be a signal of the progression of disease from CML to blastic crisis. In patients without a history of myeloproliferative disorders, granulocytic sarcoma is usually an ominous sign. Reports in literature describe a lag period of 10–49 months between the diagnosis of MS and the development of AML [7].They are common in children with a peak prevalence in age group of 7–8 years with slight male predominance. The organs frequently involved are bones and periosteum. From the marrow, the tumor cells pass through the Haversian canals and reach the periosteum; later, they pass into the bloodstream and can reach any other organ. The other common sites involved by MS include soft tissue, lymph nodes, gastrointestinal tract, pericardium, bronchus, bladder, mediastinum, kidney, and lung.In the head and neck region, the orbit is the most preferred site followed by skull and epidural spaces. Frank granulocytic sarcoma of the temporal bone is rare [8].

On CT scans, MS are generally homogeneously isoattenuating to slightly hyperattenuating. On post contrast studies they exhibit uniform homogenous enhancement.When the bone is affected, granulocytic sarcoma lesions appear lytic rather than sclerotic [3]. MR imaging helps in better characterization of the soft tissue components. These are isointense to hypointense on T1-weighted imaging. On T2-weighted images they are heterogeneously isointense to slightly hyperintense. It is, however, not possible to distinguish MS from lymphoma, meningioma, or pseudotumor solely on the basis of imaging findings [4]. The pathologist remains the final arbiter. Diagnosis is comparatively easier when it arises in a setting of AML/MPS or MDS. However, in the absence of known haematological disorder, arriving at the diagnosis may be challenging. Differentials to consider in patients without history of leukemia include rhabdomyosarcoma, metastatic neuroblastoma and Langerhans cell histiocytosis. Possible differentials in a diagnosed case of leukemia include various complications of the disease, namely abscess, hematoma or development of secondary malignancy. Histopathology has a central role in diagnosing MS, and this is further strengthened by the use of IHC. Routine histological examination of the tumors shows pleomorphic infiltrate of primitive cells of varying sizes and nuclear configuration with mononucleate and granulocytic cells of variable maturity along with scattered eosinophilic myelocytes. Eosinophilic myelocytes are a useful clue to the diagnosis; however, they may not always be present. MS can be further subdivided into four groups based on the degree of maturation – blastic (predominantly myeloblasts), immature (myeloblasts and promyelocytes), differentiated (promyelocytes and mature neutrophils), and monoblastic sarcoma (monocytic precursors with large cells showing abundant cytoplasm). No prognostic significance is attributed to this categorization, and it has been omitted in the 2016 WHO classification of tumors of hematopoietic and lymphoid tissue [9].

Myeloid Sarcoma has to be differentiated from other morphologic mimickers which include Non-Hodgkin lymphoma (NHL) (lymphoblastic lymphoma, diffuse large Bcell lymphoma, Burkitt lymphoma), poorly differentiated carcinoma, blastic plasmacytoid dendritic cell neoplasm (BPDCN), and small round blue cell tumors such as RMS and neuroblastoma. The differentials vary depending on the age of the patient and site of occurrence. IHC has an important role in this regard. CD68KP1 is the most frequently expressed marker in MS, followed by MPO, CD117, CD99, CD68/PGM1, lysozyme, CD34, terminal deoxynucleotidyl transferase (Tdt), CD56, CD61/linker of activated T lymphocyte/ factor VIIIrelated antigen, CD30, glycophorin A, and CD4. Expression of CD 117 has been associated with poorer outcomes. NHLs are positive for LCA and either Tor Bcell markers. MS may show weak LCA positivity but will be negative for B and Tcell markers. Carcinomas are generally cytokeratin positive. BPDCN shows cutaneous involvement and is positive with CD4, CD56, and CD123 [8]. CD99 positivity in MS can cause diagnostic confusion with small round blue cell tumors. Other specific markers such as desmin (RMS) are essential as observed in our cases. Fluorescent in situ hybridization and/or cytogenetic studies can detect chromosomal aberrations such as monosomy 7, trisomy 8, MLL rearrangement, inv (16), monosomy 16, 16q, 5q, 20q, and trisomy 11 in around 55% of the cases. About 16% cases show evidence of (nucleophosmin) NPM1 mutation [4].

Owing to their rarity, there are currently no guidelines on whether to begin or delay treatment in patients without the involvement of the bone marrow. It has been documented that delayed or inadequately treated isolated MS will almost always progress to AML, with a median time of 5–12 months. There exist a variety of treatment options including chemotherapy, radiotherapy (RT), surgical excision, HSCT, or any combination of these treatments. In patients with isolated MS, treatment with AML-based induction regimens had complete remission rates comparable to those with AML without MS and they prolonged disease-free survival from 3.5 to 16 years. Induction chemotherapy with cytarabine and daunorubicine has been reported to induce complete remission in 65%–75% of patients. Radiotherapy could be used for consolidation along with systemic chemotherapy. A recent study suggested that RT may prolong failure-free survival but not overall survival in patients presenting with isolated MS. Surgery could be an option for tumors causing organ dysfunction and/or obstruction. However, systemic therapy should be considered in all patients as soon as the diagnosis is confirmed to prevent or prolong relapse and progression to acute leukemia [10]. The mean survival time has been reported to be from 2.5 to 22 months. Hematopoietic stem cell transplantation can prove useful in patients in remission or relapse following chemotherapy. Prognosis varies with the clinical situation. This case illustrates the challenges associated with developing a rapid diagnosis with early initiation of treatment.

## 4 Conclusion

MS are extremely rare tumors with an aggressive clinical course. Morphologically, they can mimic small round cell tumors. This problem is further complicated by the lack of clinical suspicion in patients without any hematological disorder. Accurate diagnosis of isolated MS requires a multifactorial approach including histopathology, immunophenotyping, immunohistochemistry and cytogenic abnormalities. A high level of suspicion is necessary, because early, accurate diagnosis is important to avoid delaying appropriate chemotherapy. A delay in the diagnosis may result in unwarranted fatality particularly so in pediatric patients.

## Conflicts of interest

We have no conflict of interest.

## Sources of funding

Nil.

## Ethical approval

Ours is a single case report, therefore, it was exempted by the ethics committee.

## Consent

Written informed consent was obtained from the patient’s parents for publication of this case report and accompanying images. A copy of the written consent is available for review by the Editor-in-chief of this journal on request.

## Author’s contribution

Study concept: Dr. Nisha Marwah.

Writing the paper: Dr. Namita Bhutani.

Data interpretation: Dr. Rajeev Sen.

Pictures: Dr. Archana Budhwar.

## Registration of research studies

Not applicable.

## Guarantor

Dr. Namita Bhutani.

## Provenance and peer review

Not commissioned, externally peer-reviewed.
